# Assessing Population Trends of Species with Imperfect Detection: Double Count Analyses and Simulations Confirm Reliable Estimates in Brown Frogs

**DOI:** 10.3390/ani12162085

**Published:** 2022-08-15

**Authors:** Mattia Falaschi, Chiara Gibertini, Elia Lo Parrino, Martina Muraro, Benedetta Barzaghi, Raoul Manenti, Gentile Francesco Ficetola

**Affiliations:** 1 Department of Environmental Science and Policy, University of Milan, Via Celoria 10, 20133 Milan, Italy; 2 Laboratoire d’Écologie Alpine, University Grenoble Alpes, University Savoie Mont Blanc, CNRS, LECA, F-38000 Grenoble, France

**Keywords:** egg clutch detectability, multinomial *N*-mixture models, population size, *Rana dalmatina*, *Rana latastei*

## Abstract

**Simple Summary:**

Estimating the abundance of populations is a key task of zoologists, but animal species often are difficult to detect- As a consequence, obtaining estimates of abundance can be problematic. For many frogs, the count of the number of egg clutches is the standard approach. How many egg clutches are missed during these counts? What are the consequences of missing some egg clutches on the estimates of population decline? First, we used the double-observer approach to estimate the detection probability of the clutches of two frog species (the agile frogs *Rana latastei* and *R. dalmatina*) and found that, for both species, clutches are very easy to detect. Subsequently, by using simulations, we showed that if the detection probability is very high, such as in this case, assessing if populations are stable or declining is straightforward. The situation is much trickier for species that are difficult to detect. Information on the detection probability of a species can be used to optimize their monitoring strategies.

**Abstract:**

Most animal species are detected imperfectly and overlooking individuals can result in a biased inference of the abundance patterns and underlying processes. Several techniques can incorporate the imperfect detection process for a more accurate estimation of abundance, but most of them require repeated surveys, i.e., more sampling effort compared to single counts. In this study, we used the dependent double-observer approach to estimate the detection probability of the egg clutches of two brown frog species, *Rana dalmatina* and *R. latastei*. We then simulated the data of a declining population at different levels of detection probability in order to assess under which conditions the double counts provided better estimates of population trends compared to naïve egg counts, given the detectability of frog clutches. Both species showed a very high detection probability, with average values of 93% for *Rana dalmatina* and 97% for *R. latastei*. Simulations showed that not considering imperfect detection reduces the power of detecting population trends if detection probability is low. However, at high detection probability (>80%), ignoring the imperfect detection does not bias the estimates of population trends. This suggests that, for species laying large and easily identifiable egg clutches, a single count can provide useful estimates if surveys are correctly timed.

## 1. Introduction

Every wildlife biologist has experienced the frustration of being unable to find any individual of his target species during a field survey, even in habitats where a colleague or a friend has reported several sightings of that species. This can occur because animals are not available in a given moment at a study site, or because the individuals are present but their detection probability is lower than one [1,2], which may depend on their behavior and characteristics, on the environmental conditions at the survey sites, or on the ability level of an observer to detect the individuals in a given area [3,4,5]. Overlooking individuals during counts at a site can result in biased estimation of species occurrence and abundance, as well as to misleading inferences on the key parameters of populations or of the relationships between species and habitats. For instance, not considering detection probability can lead to strongly biased estimates of the effect of environmental features on a species’ presence or abundance [6]. Furthermore, overlooking an imperfect detection process causes a systematic underestimation of species distribution and abundance across a study area [1]. Knowing the number of individuals in a population and how this abundance changes over time is the most direct way to understand where conservation efforts are most needed and what are the processes determining a population’s decline [7,8,9]. In fact, many assessments of species conservation (e.g., the IUCN Redlist or the reporting of species listed under the European Union Habitat Directive) consider the abundance of populations as a key parameter to evaluate the conservation status of an assessed species [10].

The imperfect detection of a target species is also a main issue when estimating trends of population abundance. In fact, if a species has a low detection probability, it becomes challenging to estimate whether its populations are stable or declining [10]. Additionally, detection probability can vary across study sites and over time, increasing the risk of biased inference [11,12,13]. As a result, repeated surveys are often required, should we want to obtain reliable trend estimates, particularly when detection probability is low [10], while a species with a high detection probability is less impacted by these issues. For instance, simulations suggested that the ability to correctly estimate population trends is higher at higher values of detection probability [10]. On the other hand, performing multiple surveys is costly and conservation resources are generally limited, and thus it is important to optimize the survey efforts. Knowing the actual detection probability is therefore extremely important for evaluating how many surveys are needed for a correct assessment of population abundance and of a species’ conservation status.

The dependent double-observer approach is a promising method to rapidly estimate the actual abundance of species and has been successfully applied to several amphibian species [3,14]. Previous studies on American amphibians (*Ambystoma maculatum*, *Rana sylvatica*) in the United States showed the high detectability of egg clutches [14]. However, to our knowledge, no study is available on the detectability of egg clutches of brown frogs in Europe, for which clutch counts are often the basis for a wide range of ecological and conservation studies and for the assessment of temporal trends [15,16,17,18,19,20]. Furthermore, there have been limited assessments of how much imperfect detection can bias estimates of a species’ trend. In this study, we performed dependent double-observer counts of egg clutches of two brown frog species occurring in northern Italy (*Rana dalmatina* and *Rana latastei*) to estimate the detection probability of egg clutches. Then, we ran simulations to evaluate the effect of not considering the detection process in analyzing population trends of abundance. We simulated species with different levels of detection probability and different average population sizes, with the aim of understanding under which conditions the use of approaches such as double counts are necessary to avoid biased estimates of population trends.

## 2. Materials and Methods

### 2.1. Study System

**Figure 1:**
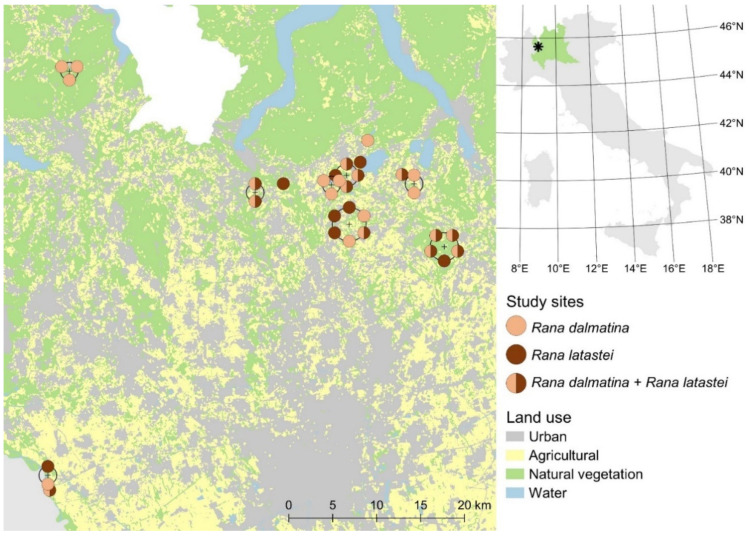
Location of the 32 studied wetlands in northern Italy, with indication of the sites where egg clutches of *Rana dalmatina* (light brown) and *Rana latastei* (dark brown) were detected. The position of areas with multiple overlapping wetlands at this scale is indicated by crosses, and the dots indicating species presence are placed on circles surrounding these crosses. The position of the Lombardy region in Italy is highlighted in green in the top-right inset and the location of the study area is indicated by a black asterisk.

The study area consists of 32 ponds, ditches, and lakeshores (hereafter: wetlands), located in the northwestern Lombardy region of northern Italy (Figure 1). The studied wetlands are breeding sites for several amphibian species, including two brown frogs: the agile frog, *Rana dalmatina*, and the Italian agile frog, *Rana latastei*. The two study species are medium-sized frogs which are mostly terrestrial and spend only the short reproductive period in an aquatic environment, corresponding to late winter/early spring [21]. Both species lay globular egg masses that can contain several hundreds or even thousands of eggs (600–2000 eggs for *R. dalmatina* and 500–2500 eggs for *R. latastei*), and each female lays a single egg clutch per year; hence, a precise count of the number of egg clutches corresponds to the number of breeding females in a given wetland [17,22].

Between late winter and early spring 2022, we performed double counts of egg clutches at the study sites separately for the two species. The surveys were timed to spot the peak of the breeding season of the two frogs and, during the sampling, the two observers walked across the entire wadable surface of the wetlands to count the clutches of the two species. We adopted a dependent double-observer approach, where a first observer indicates clutches to a second observer, who annotates the number of clutches not seen by the first observer [3,14]. Before beginning the fieldwork, we performed preliminary independent counts in a small subset of ponds and obtained identical counts from the different observers. Hence, we assumed that their ability was comparable. All the clutch counts for the two observers are available in Table A1.

### 2.2. Estimating Detection Probability of Egg Clutches

To estimate the detection probability of egg clutches, we used hierarchical dependent double-observer models, which are hierarchical models that estimate abundance by taking into account the imperfect detection of the study species [23]. We ran a separate model for each species, assuming that the counts were distributed following a Poisson distribution with a log link. Models were built using the “multinomPois” function of the R package unmarked [24] and the “type = depDouble” argument, which is used to analyze dependent double-observer counts. We ran a separate model for each species to estimate the average detection probability of the egg clutches.

### 2.3. Simulation of Population Declines

To test the possible effect of not considering the detection probability on the estimation of population trends, we analyzed simulated data. We simulated single counts of a model organism across 50 study sites over 5 years, assuming a 2% decline per year. To simulate a situation that was as real as possible, the decline was not constant across all the study sites, but each site followed a decline drawn from a normal distribution, with a mean = 0.02 and a standard deviation = 0.001. As a consequence, some study sites declined slightly more and others declined slightly less than the average 2% yearly decline. The true abundance *N* in the first year across all study sites followed a Poisson distribution with mean *L*. We simulated three cases with an average population size (*L*) of 30, 60, and 90 individuals. For each case, we then simulated nine different datasets, each with a constant detection probability (*p*) varying from 0.10 to 0.90 by 0.10 increases. To simulate the imperfect detection process, the observed abundance at each site (*y*) followed a binomial distribution:

$$y_{i,t}=Binomial\left(N_{i,t},\;p_{d}\right)\;$$

where *y_i,t_* is the observed abundance at site *i* in year *t*, *N_i,t_* is the true abundance at site *i* in year *t*, and *p_d_* is the detection probability for the dataset *d*. In so doing, we simulated 27 datasets, each with 1000 replicates, corresponding to nine levels of detection probability × three levels of average population abundance. 

We analyzed the simulated datasets using generalized linear mixed models with a Poisson error distribution (log link). For each dataset, the observed abundance was the dependent variable, while the year was considered as a fixed effect in order to estimate the total decline. For each realization, we assessed whether the mixed model detected a significant decline in species abundance. Site identity was used as a random factor to account for the variation of abundance across different sites. We deliberately used a method that did not account for imperfect detection to evaluate the possible effect of not considering the detection probability for species that are detected imperfectly.

We repeated the simulations with a trend of 0% to calculate the type 1 error rate (frequency of finding a trend when it does not actually occur) and to evaluate if it changed with different detection probabilities.

## 3. Results

**Figure 2:**
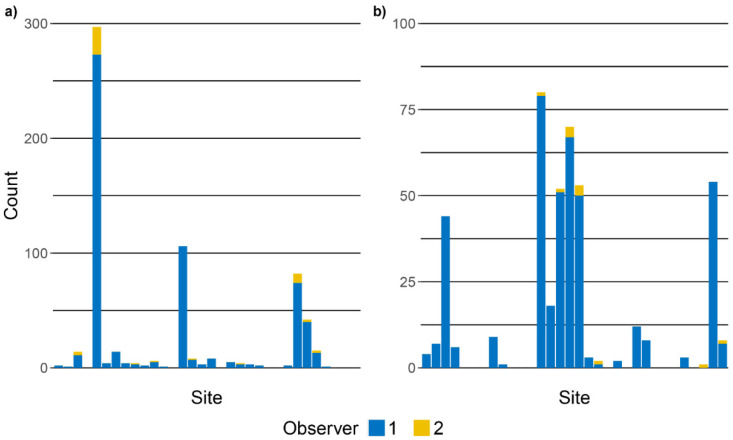
Counts of egg clutches at the 32 study sites for (**a**) *Rana dalmatina* and (**b**) *Rana latastei*. The blue bars indicate clutches detected by the first observer, and the yellow bars represent clutches not seen by the first observer and detected by the second observer.

Across the 32 study sites, we found *Rana dalmatina* clutches at 24 sites and *Rana latastei* clutches at 20 sites, with 12 sites being occupied by both species (Figure 1). The total number of clutches detected at occupied sites by the two observers ranged from 1 to 297 for *Rana dalmatina* (Figure 2a) and from 1 to 80 for *Rana latastei* (Figure 2b).

### 3.1. Detection Probability of Egg Clutches

Egg clutches for both species showed a very high detection probability: the average detection probability for *Rana dalmatina* egg clutches was 92.7% (95% confidence interval (CI) = 90.1–94.7%), while the detection probability for *Rana latastei* clutches was 97.4% (95% CI = 95.3–98.6%). The total abundance estimated by the *N*-mixture models across all study sites was, on average, 633 for *Rana dalmatina* and 437 for *Rana latastei*, respectively corresponding to 92.7% and 97.5% of the clutches detected by the first observer for the two species.

### 3.2. Analyses of Simulated Population Trend Data at Different Levels of Detection Probability

**Figure 3:**
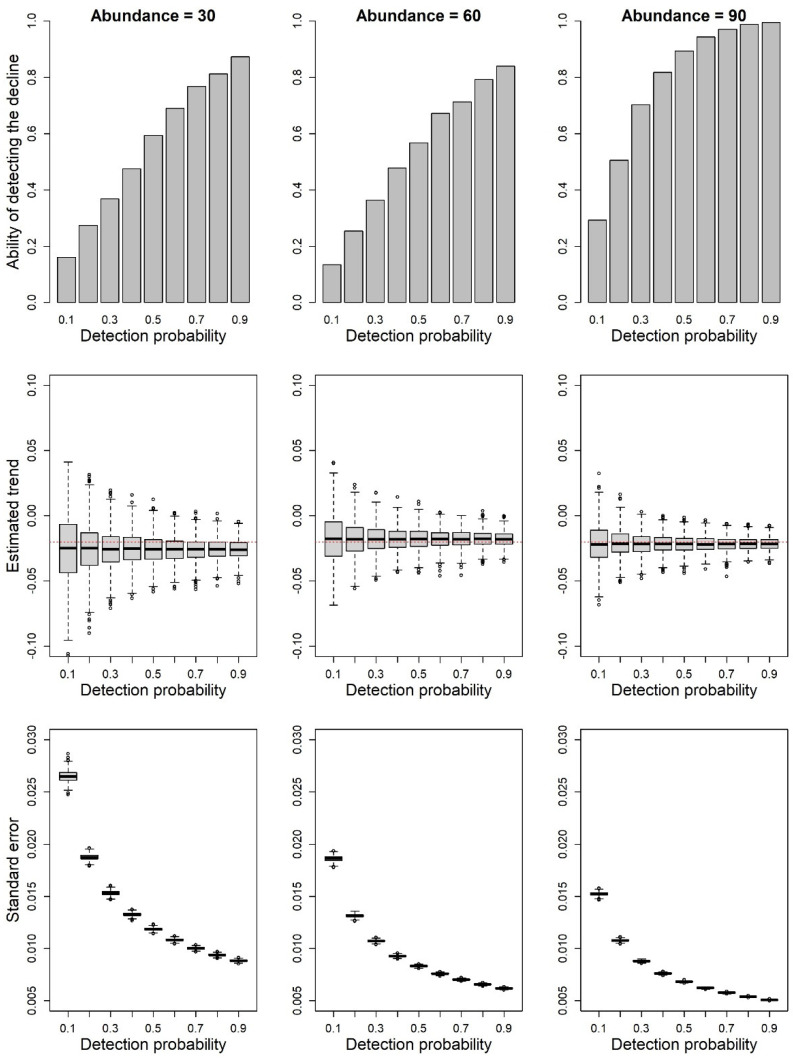
Results of the simulations estimating the assessment of decline in species with different detection probabilities. In the first row of plots, the bars represent the proportion of simulations detecting a significant (α = 0.05) decline of the target species. The actual average population size of populations (*L*) ranged from 30 to 90. In the second row, the boxplots show the distribution of the estimated trends across the 1000 simulation replicates. The red line represents the actual trend. In the third row, the boxplots show the distribution of the standard errors of the estimated trends across the 1000 replicates.

The simulated data showed that the power of detecting population decline increases at high levels of detection probability (Figure 3). The ability of detecting a negative population decline, with a significance level α = 0.05, was lower if the average population size was small (L = 30–60) compared to situations with larger average population size (L = 90). In general, analyses of the data simulated with a detection probability of 0.9 always showed an excellent ability to detect the species decline, while if the detection probability was very low (0.1), the models generally failed at detecting the decline (Figure 3).

**Figure 4:**
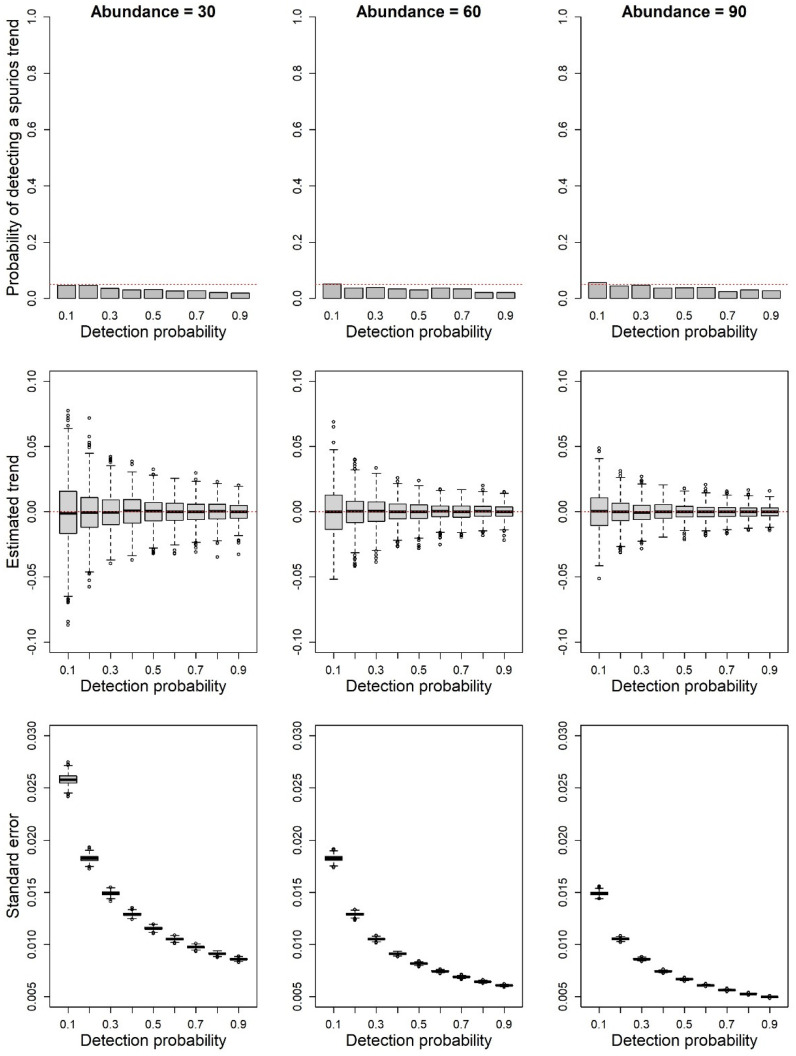
Results of the simulations estimating the assessment of trends in species with different detection probabilities (simulations assuming populations with stable trends). In the first row of plots, the bars represent the proportion of simulations detecting a significant (α = 0.05) decline or increase of the target species. The actual average population size of the populations (*L*) ranged from 30 to 90. The red line represents the expected type I error (0.05). In the second row, the boxplots show the distribution of the estimated trends across the 1000 simulation replicates. The red line represents the actual simulated trend. In the third row, the boxplot shows the distribution of the standard errors of the estimated trends across the 1000 replicates.

The models analyzing simulations with stable populations only rarely detected a positive or negative trend (Figure 4). The frequency of the models detecting spurious trends was very low and it decreased in cases with high detectability (*p* = 0.8–0.9). In these cases it was always lower than α = 0.05.

## 4. Discussion

By performing dependent double-observer counts of egg clutches of *Rana dalmatina* and *Rana latastei*, we found a very high detection probability for both species. In our field study, only a small portion of the egg clutches were only detected by the second observer (Figure 2) and the first observer was able to detect > 90% of the clutches estimated by the double-observer models for both species. The high detectability of brown frog clutches is consistent with other frog species from North America [14], suggesting that for species laying large and easily identifiable egg clutches, a single count per season might be enough to assess population trends, if the surveys are correctly timed.

With double-observer counts, we assessed the detectability of clutches that were available in a given moment, making no inference on the patterns of intra-annual variation at the study sites. However, many amphibians can show complex patterns of seasonal migration outside and inside breeding wetlands. In these cases, a correct timing of the surveys is fundamental, and it is often impossible to obtain accurate measures of abundance with a single survey, requiring multiple intra-annual site visits to study population dynamics [25,26,27,28,29]. In the case of brown frog populations, the correct timing of surveys can be achieved through preliminary monitoring of a selected subset of sites in late winter. These species show an explosive breeding pattern, with adults exploiting wetlands only for a short period [30]. Hence, it is crucial to plan egg clutch counts during the 2–4 weeks’ time window before hatching, when clutch abundance at breeding sites reaches a peak, in order to avoid a strong underestimation of breeding females.

By analyzing simulated data, we demonstrated that models not accounting for detection probability can well estimate population trends if the target species has a very high detectability (Figure 3), while estimates can be much more uncertain for the least detectable species. Furthermore, the necessity of using survey methods and models enabling taking into account detection probability does not depend only on the detection level of a given species. In fact, by assessing the ability to detect trends in sets of populations with three different levels of average abundance, we showed that models not accounting for imperfect detection have a better power and give more consistent results for abundant populations (Figure 3). This confirms the finding of other studies that showed the need for a high sampling effort, including a large number of either sites, or surveys per site, to detect population trends when the abundance of the study species is low [10]. Another important aspect that can influence the ability of models to detect population declines is the strength of the decline, with fast declines being easier to detect than slow declines [10]. Here, we used a 2% decline each year (totaling approximately a 10% decline over five years), which can be considered a moderate decline and is possibly concerning from a local conservation perspective. In our simulations, models consistently identified the decline only at high levels of detection probability, suggesting that repeated surveys and models accounting for detection probability can be particularly useful when target species have a detectability of <80%.

Additional factors can influence our ability to estimate the correct population trend, as detection probability might be not constant over space or time. For instance, phenology, survey-specific meteorological conditions, or some breeding site characteristics can determine variations in detectability [4,31]. In our simulations, we assumed a constant detection probability across the study sites and over time. In principle, strong changes in detection probability could affect trend estimates. In cases where detection probability is not constant and surveys are performed, for instance, under different meteorological conditions, we expect a bias in models not accounting for the heterogeneity of detection probability. Previous studies have suggested that the heterogeneity of detection probability has a limited impact on the estimation of population trends [10], still additional studies are required to evaluate the potential impacts of systematic variations of detection probability. In any case, when detection probability is very high, estimates of population trends from single counts should produce reliable results with a low bias, high precision (Figure 3), and limited type I errors (Figure 4).

## 5. Conclusions

The detection probability of egg clutches of brown frog species, such as *Rana dalmatina* and *Rana latastei*, is very high. This suggests that single counts might be enough to appropriately evaluate population trends of these species, even though counts should be correctly timed with respect to the species’ reproductive phenology. In these cases, the resources available for monitoring can be directed to monitoring the largest number of sites in order to better estimate the situation over broad spatial scales. In any case, the application of sampling designs and models that take into account the imperfect detection process remains essential for an unbiased inference of population dynamics for species with lower detection probabilities, allowing a more precise estimation of temporal changes for conservation purposes [10,25].

## Data Availability

All data are available in Appendix A.

## Appendix A

**Table A1:** Dataset used for the analyses of dependent double-observer counts of brown frog clutches. “Randal” and “Ranlat” indicate the counts for *Rana dalmatina* and *Rana latastei* for the first and second observers. Empty cells indicate sites with no counts of the target species.

Site	Randal1	Randal2	Ranlat1	Ranlat2
1			0	1
2	1	0		
3	13	2	3	0
4	2	0		
5	5	1		
6	1	0		
7	2	0		
8			8	0
9	5	0	1	1
10	74	8		
11	40	2		
12			3	0
13	4	0	9	0
14	3	1	1	0
15	14	0		
16	4	0		
17	273	24		
18	11	3	44	0
19	1	0	7	0
20			6	0
21	2	0	4	0
22	8	0	50	3
23	3	0	67	3
24	7	1	51	1
25	106	0	18	0
26			79	1
27	3	1		
28	3	0	2	0
29	2	0		
30			12	0
31			54	0
32			7	1
